# Investigation of molecular regulation mechanism under the pathophysiology of subarachnoid hemorrhage

**DOI:** 10.1515/biol-2021-0138

**Published:** 2021-12-31

**Authors:** Yifei Weng

**Affiliations:** Department of Neurology, The Affiliated People’s Hospital of Ningbo University, No. 251 East Baizhang Road, Ningbo City, Zhejiang Province, 315040, People’s Republic of China

**Keywords:** subarachnoid hemorrhage, RNAs, support vector machine classifier, biomarkers

## Abstract

This study aimed to investigate the molecular mechanism under the pathophysiology of subarachnoid hemorrhage (SAH) and identify the potential biomarkers for predicting the risk of SAH. Differentially expressed mRNAs (DEGs), microRNAs, and lncRNAs were screened. Protein–protein interaction (PPI), drug–gene, and competing endogenous RNA (ceRNA) networks were constructed to determine candidate RNAs. The optimized RNAs signature was established using least absolute shrinkage and selection operator and recursive feature elimination algorithms. A total of 124 SAH-related DEGs were identified, and were enriched in inflammatory response, TNF signaling pathway, and others. PPI network revealed 118 hub genes such as *TNF*, *MMP9*, and *TLR4*. Drug–gene network revealed that chrysin targeted more genes, such as *TNF* and *MMP9*. *JMJD1C*-*AS*-*hsa*-*miR*-*204*-*HDAC4*/*SIRT1* and *LINC01144*-*hsa*-*miR*-*128*-*ADRB2*/*TGFBR3* regulatory axes were found from ceRNA network. From these networks, 125 candidate RNAs were obtained. Of which, an optimal 38 RNAs signatures (2 lncRNAs, 1 miRNA, and 35 genes) were identified to construct a Support Vector Machine classifier. The predictive value of 38 biomarkers had an AUC of 0.990. Similar predictive performance was found in external validation dataset (AUC of 0.845). Our findings provided the potential for 38 RNAs to serve as biomarkers for predicting the risk of SAH. However, their application values should be further validated in clinical.

## 1 Introduction

Intracranial aneurysm (IA) is one of the common neurological diseases, and its incidence rate in the general population is approximately 5% [1]. IA is characterized by localized dilation or ballooning of a cerebral artery. Once an IA ruptures, a subarachnoid hemorrhage (SAH) typically develops [2,3]. SAH is a severe subtype of stroke, occurring in people about 50 years old [4]. Previous research revealed that environmental exposures and genetic predisposition play a role in the susceptibility of SAH, and the estimated heritability is about 40% [5]. Recently, despite considerable advances in therapy for IAs, SAH remains a highly challenging condition associated with a high socioeconomic burden [6,7]. SAH is a critical disease that has to be treated immediately. Therefore, an in-depth understanding of the molecular mechanism of SAH is necessary for the treatment of SAH. In addition, early screening and early active management and prevention of SAH help to reduce the mortality and disability rate of SAH patients. For these two purposes, this study was designed to investigate the molecular mechanism under the pathophysiology of SAH and to identify potential biomarkers that could predict the risk of SAH.

With the development of bioinformatics, gene expression profiling has been widely used to identify the biomarkers for the diagnosis and treatment of SAH [8]. Wang et al. found that six hub genes, *BASP1*, *CEBPB*, *ECHDC2*, *GZMK*, *KLHL3*, and *SLC2A3*, were determined as biomarkers to assess the progression and rupture of IAs [3]. It is known that long non-coding RNAs (lncRNAs) interact with mRNAs, and microRNAs (miRNAs) regulate many processes, such as transcription, translation, regulation of cell differentiation and cell cycle [9]. Interestingly, non-coding RNAs, comprising miRNAs and lncRNAs, play an important role in IAs and SAH [10]. Besides, lncRNAs detected from the biological fluids may be used as non-invasive biomarkers for the diagnosis and prognosis of IAs and SAH [11]. For instance, lncRNA *MALAT1* expression was independently associated with the poor overall survival for IAs, and the overexpression of *MALAT1* predicted an higher risk of death in IA patients [12]. Circulating miRNAs (such as *miR-16* and *miR-25*) may be novel biological markers that are useful in assessing the likelihood of IA occurrence [13]. Unfortunately, because of poor understanding of the mechanisms of SAH, current diagnosis and treatment of SAH can be inconsistent and/or ineffective [14,15]. Especially, the effects of core RNAs on the progression and prognosis of SAH patients have not been fully identified.

**Figure 1:**
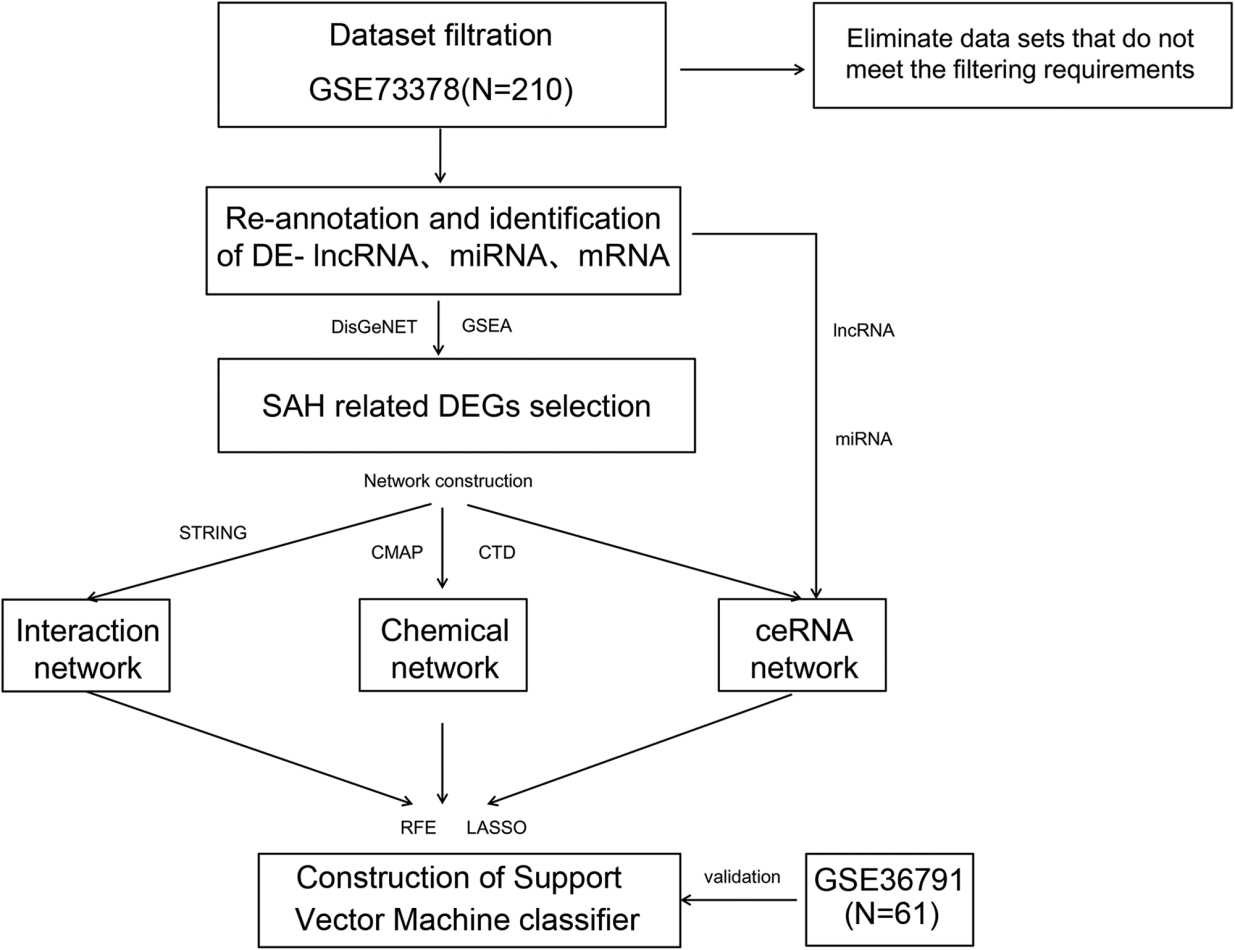
Flow diagram of the present study.

In the present research, we aimed to screen the SAH-related RNAs as biomarkers to provide new insights for the early screening, diagnosis, and treatment of SAH. For this aim, GSE36791 [16] and GSE73378 [15] datasets from the Gene Expression Omnibus (GEO) database were reanalyzed. A flowchart presenting the experimental design of this study is illustrated in Figure 1.

## 2 Materials and methods

### 2.1 Data collection and preprocessing

The microarray datasets searched by terms of “subarachnoid hemorrhage” and “*Homo sapiens*” were acquired in the GEO database as of 2 January 2021. For the purpose of this research, the dataset screening criteria were as follows: (1) blood samples; (2) samples of SAH patients and controls; and (3) the total number of samples >50. There were two datasets meeting the screening criteria, GSE73378 [15] and GSE36791 [16] datasets. The GSE73378 dataset had a total of 226 samples, of which 210 blood samples including 103 SAH samples and 107 control samples were analyzed in this study. GSE36791 dataset had a total of 61 blood samples including 43 SAH samples and 18 control samples. The platform of these two datasets was GPL10558 Illumina HumanHT-12 V4.0 expression beadchip. The corresponding platform annotation files were downloaded from Ensembl genome browser 96 database. Then, the probes in the two datasets were annotated to lncRNAs, miRNAs, and mRNAs based on the annotation files. Of the two datasets, all the analyses were performed based on GSE73378 dataset, and GSE36791 was used just for validation of the expression and predictive performance of the selected feature RNAs.

### 2.2 Assessment of differentially expressed RNAs

In GSE73378 dataset, the differentially expressed mRNAs (DEGs), miRNAs (DEMs), and lncRNAs (DELs) from SAH samples and normal samples were analyzed using the limma package (Version 3.34.7) [17] in R 3.6.1 language. The cutoff for differentially expressed RNAs (RNAs) screening was a false discovery rate (FDR) < 0.05 and |log_2 _FC| > 0.263 (1.2 fold changes). Euclidean distance-based two-way hierarchical clustering analysis [18,19] was performed for the identified DERs using the pheatmap package (Version 1.0.8) [20] in R 3.6.1 language.

### 2.3 SAH-related DEGs screening and functional enrichment analysis

SAH-associated genes were downloaded from DisGeNET database [21] by term of “subarachnoid hemorrhage.” The SAH-associated genes from DisGeNET database were used as the reference gene sets, gene set enrichment analysis was performed for all genes detected in GSE73378 dataset (genes were ranked by corresponding log FC value) using GSEA software (http://software.broadinstitute.org/gsea/index.jsp) [22] to further identify SAH-associated genes from GSE73378 dataset. Then, the obtained SAH disease-related genes were merged with DEGs, and the overlapped genes were selected as the SAH-related DEGs. The SAH-related DEGs were used to perform Gene Ontology (GO) enrichment analysis (biological process) and Kyoto Encyclopedia of Genes and Genomes (KEGG) analyses with the DAVID online tool (version 6.8) [23,24]. FDR < 0.05 was considered to be significantly enriched.

### 2.4 Construction of protein–protein interaction (PPI) network

Interactions among the protein-coding genes in SAH-related DEGs were retrieved from the STRING database (Version 11.0) [25] with PPI score of 0.4. PPI network was visualized using Cytoscape software (Version 3.6.1) [26] based on interaction pairs.

### 2.5 Construction of the drug–gene network

Connectivity Map (CMap) resource was created to connect human diseases with the genes that underlie them and drugs that treat them. CMap is the first installment of a reference collection of gene-expression profiles from cultured human cells treated with small bioactive molecules, for uncovering the functional connections among diseases, genetic perturbation, and drug action [27,28]. The Comparative Toxicogenomics Database (CTD) is a public resource based on published literature, manually curated associations among genes, chemicals, phenotypes, diseases, and environmental exposures [29]. To predict the small molecule drugs that target the SAH-related DEGs, both CMap and CTD were used. First, SAH-related DEGs were searched from CMap database to obtain the drug molecule–gene interactions. Second, the SAH-related DEGs were uploaded to CTD database to obtain the drug molecule–gene interactions. Then, the overlapped drug molecule–gene interactions from the two databases were selected. Finally, the drug–gene network was visualized based on the selected drug molecule–gene interactions using Cytoscape 3.6.1 software.

### 2.6 Construction of competing endogenous RNA (CeRNA) network

The connection relationship between DELs and DEMs was constructed by the DIANA-LncBase v2 database [30], and the lncRNA–miRNA interactions with negative correlations of their expression level were selected. The DEMs-associated target genes (miRNA–mRNA) were predicted using five miRNA databases including TargetScan Version7.2 [31], picTar [32], miRanda [33], RNA22 [34], and PITA [35]. The miRNA–target gene interaction pairs were selected if they were predicted in more than three databases and were further filtered by SAH-related DEGs. Finally, the ceRNA network was established by integrating lncRNA–miRNA interactions and miRNA–mRNA interactions using Cytoscape 3.6.1 software.

### 2.7 Screening of optimal RNAs signature

All RNAs (mRNAs, miRNAs, and lncRNAs) contained in these three networks were used to screen characteristic RNAs by two different algorithms: least absolute shrinkage and selection operator (LASSO) and recursive feature elimination (RFE). In brief, R 3.6.1 lars package (Version 1.2, https://cran.r-project.org/web/packages/lars/index.html) [36] was used to perform the regression analysis to screen characteristic RNAs. The RFE algorithm in the R 3.6.1 caret package (Version 6.0-76, https://cran.r-project.org/web/packages/caret) [37] was also used to screen the optimal characteristic RNAs. Then, we compared the results of the two algorithms and selected the overlapping RNAs as the final feature RNAs signature.

### 2.8 Evaluation and validation of optimal RNAs signature

We first extracted the expression of the optimal feature RNAs from GSE73378 dataset and GSE36791 dataset. Their expression levels in SAH and normal samples were displayed. Afterward, the Support Vector Machine (SVM) from R 3.6.1 e1071 (Version 1.6-8, https://cran.r-project.org/web/packages/e1071) [38] was used to construct the SVM classifier based on the optimal feature RNAs signature (Core: Sigmoid Kernel; Cross: 100-fold cross-validation). Both GSE73378 dataset and GSE36791 dataset were used for classifier construction. Receiver operating characteristic (ROC) curve analysis was performed with R 3.6.1 pROC (Version 1.12.1, https://cran.r-project.org/web/packages/pROC/index.html) [39] to calculate the performance of the SVM classifier for SAH. The R codes used in this study have been provided in an additional file.

## 3 Results

### 3.1 Differentially expressed RNAs in SAH

**Figure 2:**
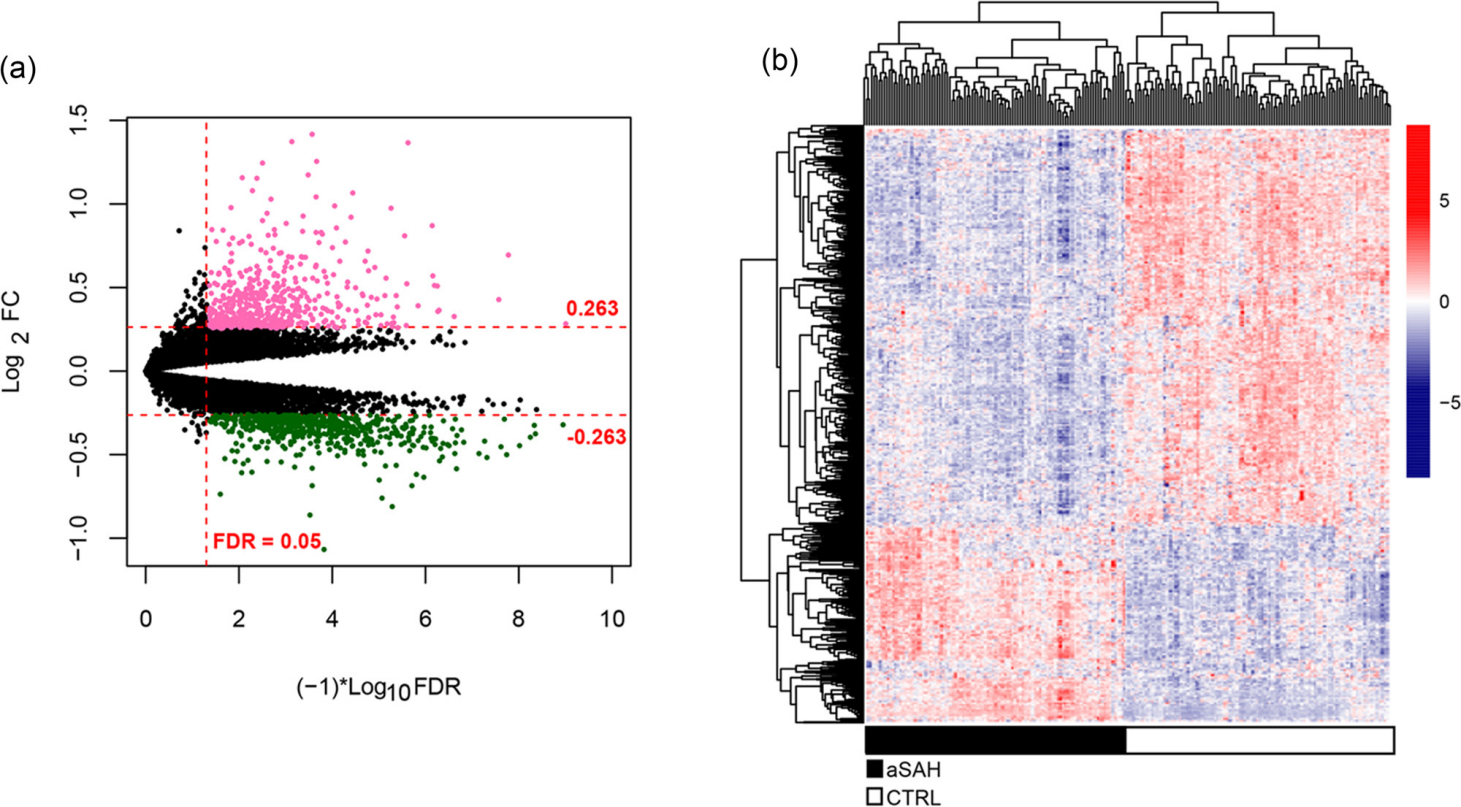
Differentially expressed RNAs analysis. (a) Volcano plot of DERs identified using the edgeR package. Red and green points indicated upregulated and downregulated DERs, respectively (|log_2 _FC| > 0.263), FC: fold change. (b) Pheatmap of DERs. Black and white color indicated the samples from patients with SAH and controls, respectively.

A total of 920 lncRNAs, 351 miRNAs, and 14,898 mRNAs were annotated. Then, 663 differentially expressed RNAs (including 17 DELs, 25 DEMs, and 621 DEGs) were identified based on the cutoff value of |log_2 _FC| > 0.263 and FDR < 0.05. Of which 228 RNAs were upregulated, and 435 were downregulated (Figure 2a). The pheatmap showed samples could be obviously distinguished into SAH and normal groups based on the differential expression level of DERs (Figure 2b).

### 3.2 SAH-related DEGs identification and function enrichment

**Figure 3:**
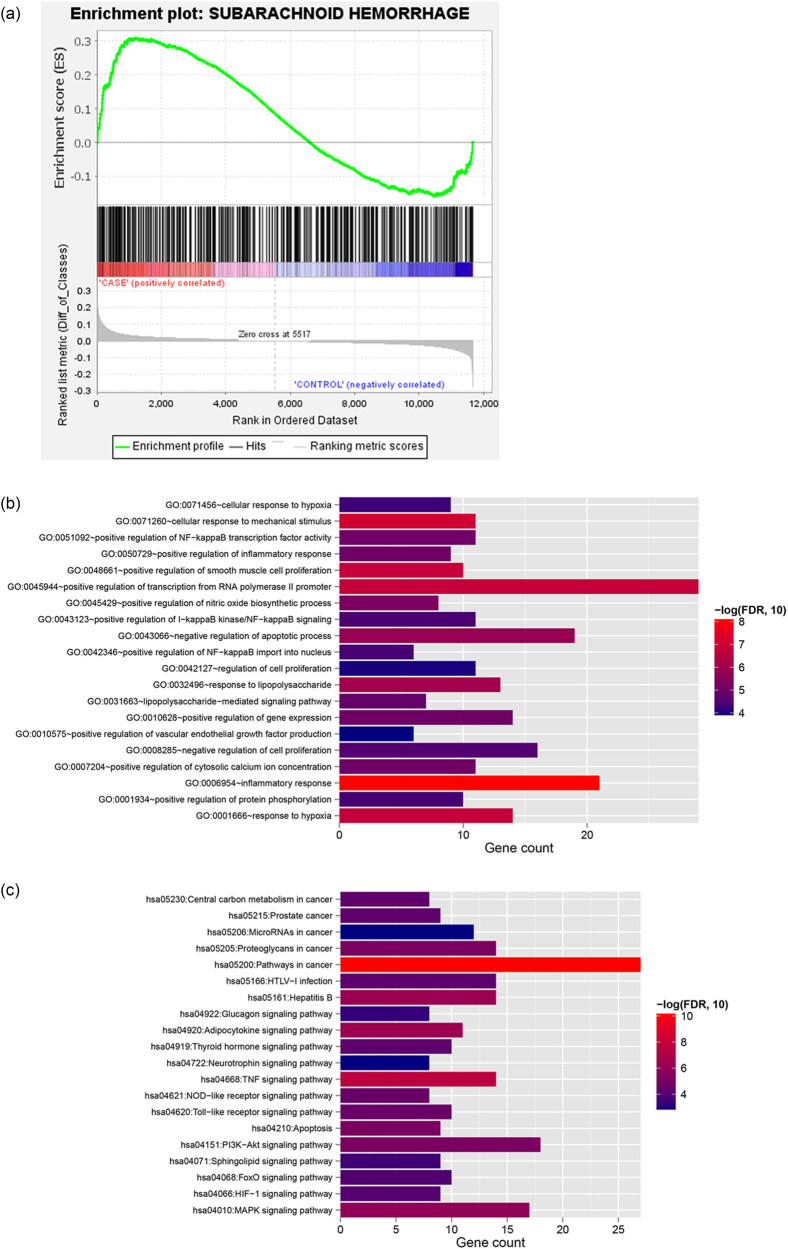
Identification of differentially expressed genes related with SAH. (a) Gene set enrichment analysis (GSEA) identified SAH-related genes. (b) The significantly enriched biological processes and (c) KEGG pathways.

From the DisGeNET database, a total of 470 genes associated with SAH were obtained. Then, GSEA was performed for all genes in SAH with the reference gene sets of SAH-associated genes from DisGeNET, and a total of 354 SAH-related genes were obtained (Figure 3a). Next the 354 SAH-related genes were compared with 621 DEGs, and a total of 124 overlapping genes were obtained as SAH-related DEGs. Enrichment analysis showed that these genes were enriched in 106 GO-biological processes, such as GO:0071260 – cellular response to mechanical stimulus, GO:0045944 – positive regulation of transcription from RNA polymerase II promoter, GO:0048661 – positive regulation of smooth muscle cell proliferation, GO:0006954 – inflammatory response, and GO:0001666 – response to hypoxia (Figure 3b). In addition, 43 KEGG pathways were significantly enriched for these genes, including hsa05200: pathways in cancer, hsa04668: TNF signaling pathway, hsa04010: MAPK signaling pathway, hsa04066: HIF-1 signaling pathway, and hsa04068: FoxO signaling pathway (Figure 3c).

### 3.3 Construction of PPI network

**Figure 4:**
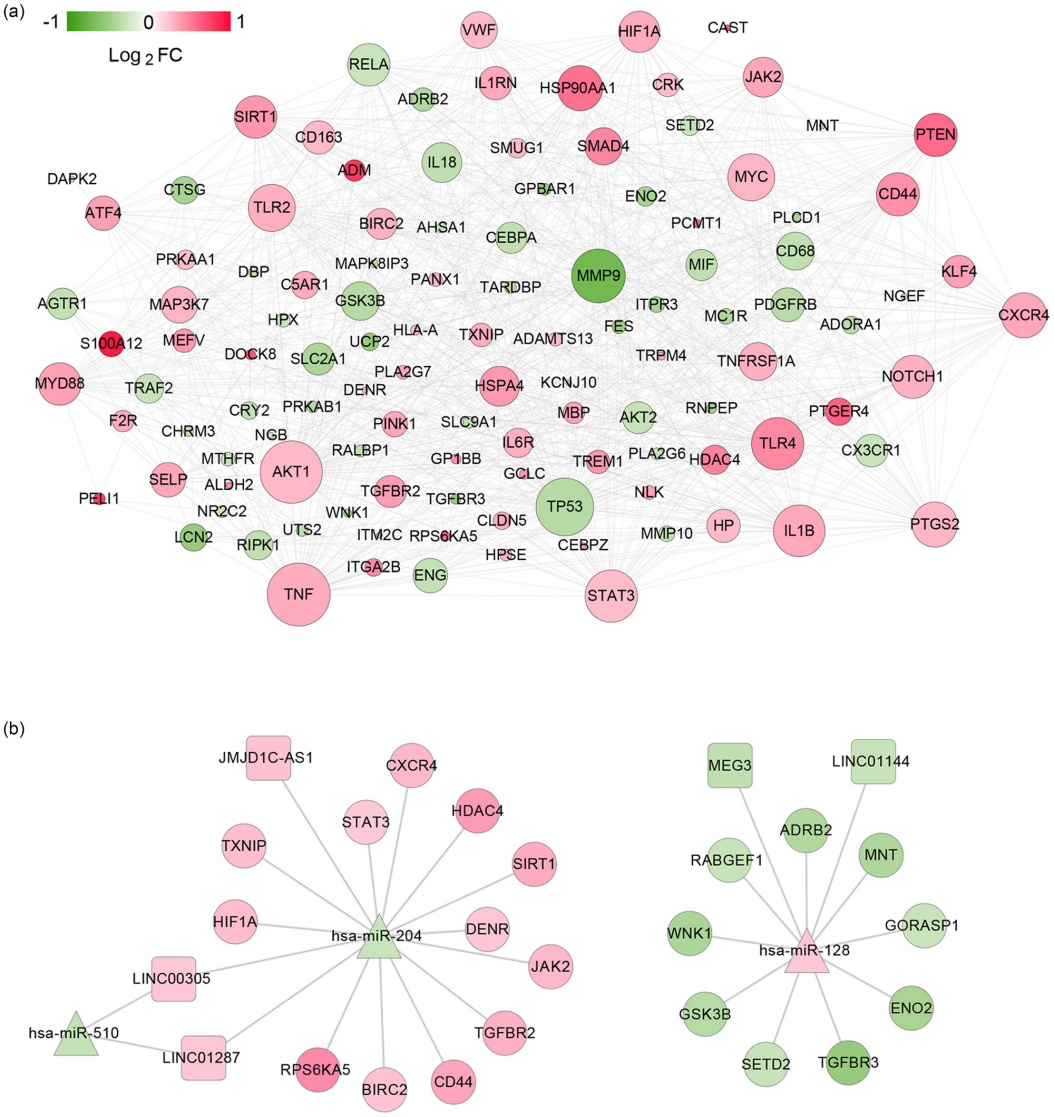
Networks construction. (a) The PPI network. The change in the color of the node from green to red indicates the change in the degree of significant difference from significantly down to up; the size of the node indicates the degree of connectivity of the node in the network. (b) The ceRNA network. Square, triangle, and circle represent lncRNA, miRNA, and mRNA, respectively. The change in the color of the node from green to red indicated the change in log_ _FC from low to high.

The SAH-related DEGs were entered into the STRING database, and a total of 830 PPI networks were generated. The PPI network, including 118 gene nodes, was constructed as shown in Figure 4a. The first ten hub genes, *TNF*, *AKT1*, *TP53*, *MMP9*, *TLR4*, STAT3, *IL1B*, *TLR2*, *MYC*, and *CXCR4,* were screened with the highest degree.

### 3.4 Construction of the drug–gene network

**Figure A1:**
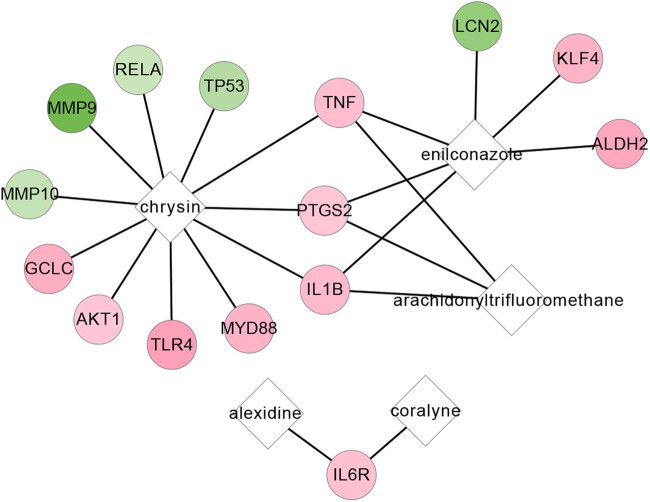
The drug–gene network. Circles and diamonds represent genes and chemicals, respectively. Red color represented the upregulated gene, and green represented a downregulated gene.

From CMAP database, a total of 23 chemicals were obtained to target SAH-related DEGs with the threshold of |Pearson *R*| > 0.75 and *P* < 0.05. The drug–gene pairs related to these 23 chemicals were further selected from the CTD database, and a total of 22 drug–gene pairs were obtained to establish a drug–gene network (Figure A1, Table A1). The network contained ten upregulated genes, five downregulated genes, and five small molecule drugs (coralyne, alexidine, enilconazole, chrysin, and arachidonyltrifluoromethane). Chrysin was found to target more genes, such as *TNF*, *AKT1,* and *MMP*9.

### 3.5 CeRNA network construction

Using the DIANA-LncBase v2 database, seven lncRNA–miRNA interactions involving three miRNAs and five lncRNAs with the negative correlation of their expression levels were obtained. Then, the target genes were predicted for 3 miRNAs in lncRNA–miRNA interactions, and then the target genes were filtered by SAH-related DEGs, and a total of 21 pairs of miRNA–mRNA connections were found. The ceRNA network was established via integration with lncRNA–miRNA and miRNA–mRNA interactions (Figure 4b). The ceRNA network comprised 29 nodes, including 5 lncRNAs, 3 miRNAs, and 21 mRNAs. Notably, upregulated *JMJD1C*-*AS1* may function as a ceRNA to suppress the inhibitory effects of *hsa*-*miR*-*204* on *HDAC4* and *SIRT1*, thus leading to their upregulated expression. Similarly, upregulated *MEG3* may regulate the expression of *TGFBR3* and *GSK3B* by binding to hsa-miR-128. In addition, *LINC01144* – *hsa*-*miR*-*128* – *ADRB2*/*TGFBR3* regulatory axis was found. We further performed correlation analysis for lncRNA and their associated mRNAs in ceRNA network (Table A2), and weak positive correlations were found. There was a significant positive correlation between *LINC01287* and *STAT3* (*r* = 0.35; *p* < 0.01), indicating that *LINC01287* – *hsa*-*miR*-*204* – *STAT3* was a potential important ceRNA regulatory axis.

### 3.6 Screening and verification of SAH-related RNAs

**Figure 5:**
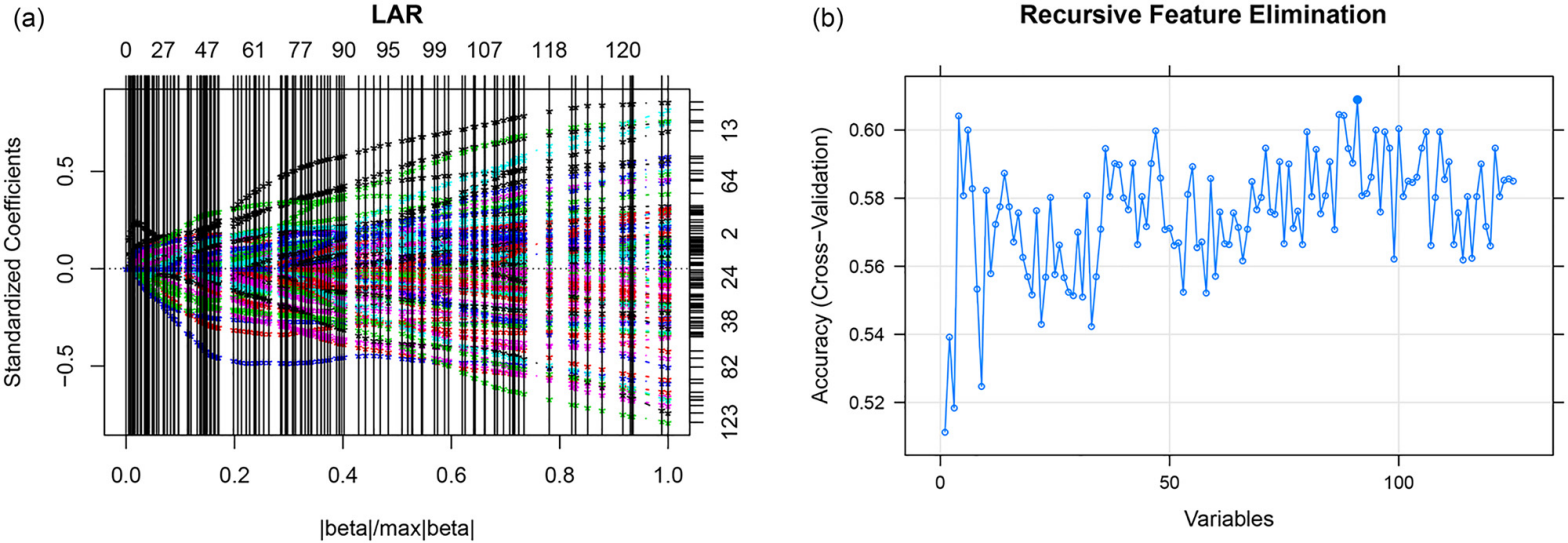
The optimal RNAs signature screened by the LASSO and RFE algorithms. (a) The standardized coefficients in LASSO algorithm; (b) cross-validation showed best accuracy at the variables of 90 in RFE.

**Figure 6:**
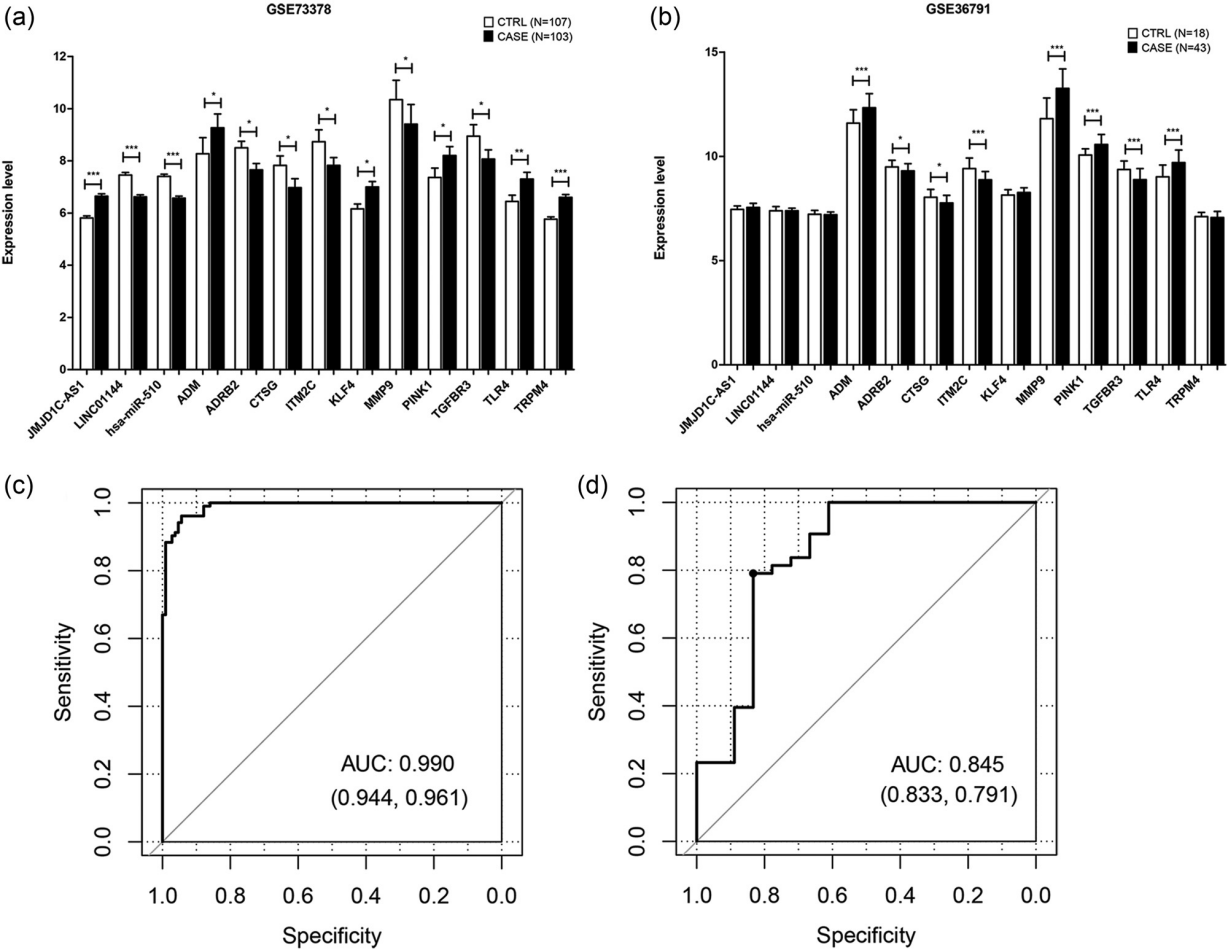
Screening and verification of SAH-related RNAs. The expression of SAH-related RNAs in the (a) GSE73378 and (b) GSE36791 datasets. *P < 0.05; 0.005 <**P < 0.05; ***P < 0.005. ROC curves for SVM classifier constructed by 38 RNAs in the (c) GSE73378 and (d) GSE36791 datasets, respectively.

LASSO and RFE algorithms were used to screen characteristic RNAs signatures from all RNAs in the three networks. In the training set (GSE73378), a total of 90 RNAs and 52 RNAs were obtained using LASSO and RFE, respectively (Figure 5a and b). Furthermore, a total of 38 overlapping RNAs were obtained as optimal characteristic RNAs signature, including 2 lncRNAs (*JMJD1C*-*AS1* and *LINC01144*), 1 miRNA (*hsa*-*miR*-*510*), and 35 genes (*TLR4*, *MMP9*, *ADRB2*, *TGFBR3,* among others) (Table 1). The expression levels of the optimal characteristic RNAs signature in SAH and normal samples are displayed in Figure 6a and b. Only the two lncRNAs, one miRNA, and top ten mRNAs (ranking by log FC) were displayed. In the GSE73378 dataset, all the 13 RNAs were significantly differentially expressed in the SAH sample compared to that of control samples (Figure 6a). While, in the GSE36791 dataset, the two lncRNAs (*JMJD1C*-*AS1* and *LINC01144*), hsa-miR-510, and mRNAs (*KLF4* and *TRPM4*) showed no statistical difference on their expression levels between SAH and normal samples (Figure 6b).

**Table 1:** The optimal 38 RNAs signature (including 2 lncRNAs, 1 miRNA, and 35 genes) screened by LASSO and RFE algorithms

ID	Symbol	Type	Log_2_ FC	*P* value	FDR	Regulation (up/down)
ILMN_1677589	JMJD1C-AS1	lncRNA	0.297887125	5.10 × 10^6^	0.004133736	Up
ILMN_1690382	LINC01144	lncRNA	−0.269639525	2.14 × 10^6^	0.00173673	Down
ILMN_3310690	hsa-miR-510	miRNA	−0.28978015	5.83 × 10^6^	0.004725453	Down
ILMN_1662809	SETD2	mRNA	−0.273252525	0.000143801	0.009318308	Down
ILMN_1666924	PINK1	mRNA	0.349209875	0.000468661	0.030369229	Up
ILMN_1671054	HLA-A	mRNA	0.27793335	0.000577726	0.037436671	Up
ILMN_1671818	UTS2	mRNA	−0.304786475	0.000176825	0.011458233	Down
ILMN_1677511	PTGS2	mRNA	0.2805209	0.000522788	0.033876642	Up
ILMN_1677532	TARDBP	mRNA	−0.3606863	9.19 × 10^5^	0.005955158	Down
ILMN_1679401	TRPM4	mRNA	0.350872575	1.62 × 10^5^	0.001051006	Up
ILMN_1680424	CTSG	mRNA	−0.425172725	0.000364996	0.023651752	Down
ILMN_1680453	ITM2C	mRNA	−0.50531015	4.88 × 10^5^	0.039519664	Down
ILMN_1680618	MYC	mRNA	0.2905886	0.000607897	0.039391751	Up
ILMN_1689734	IL1RN	mRNA	0.341397425	0.000695521	0.045069739	Up
ILMN_1695590	ADRB2	mRNA	−0.3777768	0.000218822	0.014179666	Down
ILMN_1703617	AHSA1	mRNA	−0.271606475	0.000419963	0.027213615	Down
ILMN_1706217	TLR4	mRNA	0.464493425	9.57 × 10^5^	0.006198665	Up
ILMN_1708934	ADM	mRNA	0.772645425	1.71 × 10^5^	0.013866249	Up
ILMN_1710410	CHRM3	mRNA	−0.27857305	7.68 × 10^5^	0.004974289	Down
ILMN_1715715	CEBPA	mRNA	−0.304131125	0.000418064	0.027090539	Down
ILMN_1722622	CD163	mRNA	0.27239935	0.000605937	0.039264732	Up
ILMN_1728197	CLDN5	mRNA	0.32183615	0.000479949	0.031100715	Up
ILMN_1729161	NOTCH1	mRNA	0.30529195	5.49 × 10^5^	0.044479822	Up
ILMN_1734830	MTHFR	mRNA	−0.28369415	3.74 × 10^5^	0.03025829	Down
ILMN_1748661	AKT1	mRNA	0.276206975	0.000242712	0.015727718	Up
ILMN_1760778	ENG	mRNA	−0.2935118	0.000170384	0.011040888	Down
ILMN_1779857	KLF4	mRNA	0.374529375	0.000160395	0.010393626	Up
ILMN_1783889	PRKAA1	mRNA	0.2661743	1.77 × 10^5^	0.014369739	Up
ILMN_1784287	TGFBR3	mRNA	−0.509380675	0.0001915	0.01240917	Down
ILMN_1787386	ADAMTS13	mRNA	0.2707362	4.82 × 10^5^	0.003125726	Up
ILMN_1791847	DAPK2	mRNA	−0.297182	0.000465953	0.030193776	Down
ILMN_1796180	CRY2	mRNA	−0.294307375	4.04 × 10^6^	0.003268646	Down
ILMN_1796316	MMP9	mRNA	−0.6874905	0.000225147	0.01458955	Down
ILMN_1800425	SLC9A1	mRNA	−0.319644675	0.000369868	0.023967444	Down
ILMN_1809613	NGEF	mRNA	−0.28343285	9.99 × 10^5^	0.006473637	Down
ILMN_1814327	AGTR1	mRNA	−0.29794675	9.43 × 10^5^	0.006112184	Down
ILMN_1815057	PDGFRB	mRNA	−0.338885	3.48 × 10^5^	0.002256956	Down
ILMN_2267914	CD68	mRNA	−0.305515875	0.000622021	0.040306975	Down

To validate the diagnostic ability of the optimal characteristic RNAs signature, the SVM classifier were constructed in GSE73378 dataset, which showed well predictive value for SAH patients with an AUC of 0.990 (Figure 6c). The predictive value of these optimal characteristic RNAs signature was further validated in an external independent dataset (GSE36791). The SVM classifier still showed better performance with an AUC of 0.845 (Figure 6d). The results showed that the RNAs had a robust and stable predictive ability for SAH.

## 4 Discussion

This study aimed to discover effective diagnosis biomarkers for SAH by the analysis of sequencing data, which have the potential to guide future clinical and basic medical studies. In the present research, we first identified 621 DEGs, of which 124 SAH-related DEGs were obtained using DisGeNET and GSEA. These genes were enriched in the inflammatory response, cellular response to mechanical stimulus, TNF signaling pathway, and cancer-related pathways. Increasing studies have revealed that IA is closely related to the inflammatory response [40,41]. Moreover, inflammation and immune response have also been found to potentially contribute to the formation of IA [42]. Among these pathways associated with SAH, some studies have also confirmed the role of the TNF signaling pathway in diseases including SAH. The potential of TNF-α inhibitors has been reported to impact the pathogenesis of aneurismal SAH, and the TNF-α signaling pathway has been found to play an important role in the pathogenesis of SAH [43]. In IAs, TNF-α was up-expressed in wall tissues and associated with the type and diameter of the aneurysm [44]. According to these studies, we speculated that the TNF signaling pathway was implicated in SAH development.

PPI network for SAH-related DEGs showed that *TNF*, *MMP9*, and *TLR4* were hub genes. It has been reported that venous levels of TNF-R1 were associated with poor outcomes at 6 months for SAH [45], and down-regulating *TNF*-*α* can inhibit the formation of IAs *in vivo* [44]. Thus, decreasing *TNF* expression may have the potential to inhibit SAH. *MMP9* was found to be associated with *TLR4* signaling activation, and downregulating *MMP9* induced by LPS has a neuroprotective effect on brain injury caused by SAH [46]. In addition, *TLR4* is a key player in the regulation of inflammation, and it has been found to be correlated with poor prognosis in SAH [47]. Our present results also confirmed that *TLR4* was up-expressed in SAH. Subsequently, by constructing a ceRNA network, it was suggested that the downregulated lncRNA *MEG3* may be particularly important for SAH, as it may function as a ceRNA for upregulating *hsa*-*miR*-*128* expression, thus leading to the downregulation of *ADRB2* and *TGFBR3*. Previous studies have shown that *MEG3* is highly expressed in SAH, and *MEG3* may promote SAH-induced neuronal cell injury by inhibiting the PI3K/AKT signaling pathway [48]. However, *MEG3* has also been found to promote platelet phagocytosis by decreasing *miR*-*128* expression to protect VECs from senescence [49]. To the best of our knowledge, the regulatory mechanisms of *MEG3* in SAH need further experimental confirmation. Furthermore, *TGFBR3* is involved in the activation of the *TGF*-*β* signaling pathway, and *TGFBR3* is downregulated in pancreatic ductal adenocarcinoma cells [50]. In present data, *TGFBR3* was downregulated in SAH, and overexpression of *TGRBR3* may be an important therapeutic target in SAH treatment.

We identified 38 optimal characteristic RNAs signatures from the RNAs in these networks, which were used to construct the SVM classifier. The results of ROC curves investigated that these RNAs (such as *JMJD1C*-*AS1*, *LINC01144*, *hsa*-*miR*-*510*, *TLR4*, *ADRB2*, *TGFBR3*, and so on) were potential biomarkers for predicting SAH. *MiR*-*510* has been reported to be significantly downregulated in ovarian serous carcinoma (OSC), and it is a novel candidate biomarker for predicting the symptoms of OSC [51]. However, the role of *miR*-*510* and lncRNAs, *JMJD1C*-*AS1* and *LINC01144,* in SAH has not been reported. *LINC01144*-hsa-*miR*-*128*-*ADRB2*/*TGFBR3* regulatory axis was found from our ceRNA network, *LINC01144* may play a role in SAH by regulating *ADRB2* and *TGFBR3* expression. *ADRB2* encodes adrenoceptor beta 2. Adrenoceptor polymorphisms are associated with an increased risk of cardiac abnormalities after SAH [52], β-adrenoceptor antagonists have been found to suppress the elevation of *IL-6* after SAH in rats [53]. *TGFBR3* is a transforming growth factor (TGF) beta receptor. *TGF-β1*/*Smad*/*CTGF* pathway was inhibited by rhDecorin to prevent development of hydrocephalus after SAH [54]. Knockdown of *TGF-β1* in human umbilical cord-derived mesenchymal stem cells could attenuate SAH-induced chronic hydrocephalus, upregulation of inflammatory cytokines, and other behavioral changes [55]. Considering the important role of *ADRB2* and *TGFBR3* in SAH, we speculated that *LINC01144* was involved in the development of SAH. However, elucidation of the roles of these lncRNAs associated with the screening and prevention of patients with SAH requires further investigation.

We suggested that these identified RNA biomarkers could help doctors to predict the risk of SAH and intervene as soon as possible. Although the feature RNAs were identified just based on the GSE73378 dataset, these feature RNAs still showed well predictive performance in another dataset with different inclusion criteria for patients (patients had the last episode of aneurysmal SAH at least two years in GSE73378 dataset, while this is not mentioned in GSE36791 dataset), which further indicated the stability and reliability of feature RNAs in predicting risk of SAH. Additionally, though the expression and predictive value of these feature RNAs have been validated using another external independent dataset (GSE36791), experimental verification is still indispensable in the future. In addition, the clinical value of these biomarkers should be further confirmed.

## 5 Conclusion

In summary, gene expression profile analysis revealed a large scale of expression pattern changes in RNAs under the pathophysiology of SAH, and they were mainly implicated in the inflammatory response, TNF signaling pathway. We further identified 38 RNAs, including 2 lncRNAs (*JMJD1C*-*AS1* and *LINC01144*), 1 miRNA (*hsa*-*miR*-*510*), and 35 genes (*TLR4*, *ADRB2*, *TGFBR3*, among others) as potential blood biomarkers for screening patients with SAH. This 38 RNAs signature had a better predictive performance for SAH risk. *LINC01144* might regulate *ADRB2*/*TGFBR3* expression by sponging *hsa*-*miR*-*128*. These findings of the present study contributed to understanding the molecular mechanism of SAH deeply and also provided the potential biomarkers for the screening and prevention of SAH. However, their application values should be further validated in clinical.

## Appendix

**Table A1:** The detailed information for the predicted drug–gene interactions

# Chemical name	Gene symbol	Chemical ID	Gene ID	Organism	Organism ID	Interaction	Interaction actions	PubMed IDs
Coralyne	IL6R	C000666	3570	*Homo sapiens*	9606	Coralyne results in decreased expression of IL6R protein modified form	Decreases expression	20116850
Alexidine	IL6R	C001570	3570	*Homo sapiens*	9606	Alexidine results in decreased expression of IL6R protein modified form	Decreases expression	20116850
Enilconazole	ALDH2	C017435	217	*Homo sapiens*	9606	Enilconazole results in decreased expression of ALDH2 mRNA	Decreases expression	32201337
Enilconazole	IL1B	C017435	3553	*Mus musculus*	10090	Enilconazole results in increased expression of IL1B mRNA	Increases expression	27393971
Enilconazole	KLF4	C017435	9314	*Mus musculus*	10090	Enilconazole affects the expression of KLF4 mRNA	Affects expression	29106682
Enilconazole	LCN2	C017435	3934	*Mus musculus*	10090	Enilconazole results in increased expression of LCN2	Increases expression	27393971
Enilconazole	PTGS2	C017435	5743	*Mus musculus*	10090	Enilconazole binds to and results in decreased activity of PTGS2 protein which results in decreased chemical synthesis of and results in decreased secretion of Prostaglandin D2	Affects binding|decreases activity|decreases chemical synthesis|decreases secretion	26359731
Enilconazole	TNF	C017435	7124	*Mus musculus*	10090	Enilconazole results in increased expression of TNF mRNA	Increases expression	27393971
Chrysin	AKT1	C043561	207	*Homo sapiens*	9606	Chrysin inhibits the reaction [AKT1 protein results in increased expression of MMP10 protein]	Decreases reaction|increases expression	24122885
Chrysin	GCLC	C043561	2729	*Rattus norvegicus*	10116	Chrysin dose-dependently up-regulated the protein expression of glutamate cysteine ligase (GCL) catalytic (GCLC) and modifier subunit (GCLM)	Affects binding|decreases activity|decreases reaction|increases chemical synthesis	22864849
Chrysin	IL1B	C043561	3553	*Homo sapiens*	9606	Chrysin inhibits the reaction [nickel chloride results in increased expression of IL1B protein]	Decreases reaction|increases expression	30016632
Chrysin	MMP10	C043561	4319	*Homo sapiens*	9606	Chrysin inhibits the reaction [AKT1 protein results in increased expression of MMP10 protein]	Decreases reaction|increases expression	24122885
Chrysin	MMP9	C043561	4318	*Homo sapiens*	9606	Chrysin inhibits the reaction [nickel chloride results in increased expression of and results in increased activity of MMP9 protein]	Decreases reaction|increases activity|increases expression	30016632
Chrysin	MYD88	C043561	4615	*Homo sapiens*	9606	Chrysin inhibits the reaction [nickel chloride results in increased expression of MYD88 mRNA]	Decreases reaction|increases expression	30016632
Chrysin	PTGS2	C043561	5743	*Rattus norvegicus*	10116	Chrysin inhibits the reaction [Freund’s Adjuvant results in increased expression of PTGS2 protein]	Decreases reaction|increases expression	24932515
Chrysin	RELA	C043561	5970	*Homo sapiens*	9606	Chrysin results in decreased expression of RELA protein	Decreases expression	30578657
Chrysin	TLR4	C043561	7099	*Rattus norvegicus*	10116	Chrysin inhibits the reaction [Thioacetamide results in increased expression of TLR4 mRNA]	Decreases reaction|increases expression	30500344
Chrysin	TNF	C043561	7124	*Rattus norvegicus*	10116	Silymarin promotes the reaction (chrysin inhibits the reaction [Acetaminophen results in increased expression of TNF protein])	Decreases reaction|increases expression|increases reaction	31625388
Chrysin	TP53	C043561	7157	*Rattus norvegicus*	10116	Chrysin inhibits the reaction [Testosterone results in decreased expression of TP53 mRNA]	Decreases expression|decreases reaction	29247772
Arachidonyltrifluoromethane	IL1B	C081565	3553	*Homo sapiens*	9606	Arachidonyltrifluoromethane inhibits the reaction (NAD inhibits the reaction [3′-*O*-(4-benzoyl)benzoyladenosine 5’-triphosphate results in increased secretion of IL1B protein])	Decreases reaction|increases secretion	29642561
Arachidonyltrifluoromethane	PTGS2	C081565	5743	*Mus musculus*	10090	Arachidonyltrifluoromethane inhibits the reaction [tetrachlorodibenzodioxin results in increased activity of PTGS2 protein]	Decreases reaction|increases activity	19063610
Arachidonyltrifluoromethane	TNF	C081565	7124	*Homo sapiens*	9606	Arachidonyltrifluoromethane inhibits the reaction [TNF protein results in increased expression of SOD2]	Decreases reaction|increases expression	11264281

**Table A2:** Correlation analysis results for lncRNA and their associated mRNAs in ceRNA network

DElncRNA	DEmRNA	*P* value	Cor
LINC01287	STAT3	0.002185022	0.346095443
LINC01144	RABGEF1	0.01566732	0.076406814
MEG3	ENO2	0.035022817	0.0051276
LINC00305	DENR	0.07519792	0.171671465
LINC01144	GORASP1	0.103475631	0.012136242
MEG3	GSK3B	0.115897555	0.22090058
JMJD1C-AS1	HDAC4	0.132741279	0.146195814
LINC01287	CXCR4	0.147614043	0.146372974
LINC01144	SETD2	0.154924645	0.031606782
LINC00305	CD44	0.16762594	0.030003851
MEG3	MNT	0.194071109	0.17576912
LINC00305	RPS6KA5	0.29245268	0.015048382
LINC00305	TXNIP	0.412733498	0.015264142
MEG3	GORASP1	0.414505273	0.214254126
JMJD1C-AS1	CXCR4	0.425698672	0.192223608
MEG3	ADRB2	0.439984979	0.254273266
JMJD1C-AS1	BIRC2	0.472841434	0.170991838
LINC00305	SIRT1	0.538544746	0.00332312
JMJD1C-AS1	STAT3	0.554945082	0.086126879
JMJD1C-AS1	HIF1A	0.695596063	0.035741985
MEG3	RABGEF1	0.708916655	0.175062031
LINC00305	CXCR4	0.746472986	0.199083358
LINC00305	TGFBR2	0.759578309	0.038602594
LINC00305	BIRC2	0.8264802	0.066127226
MEG3	TGFBR3	0.862145773	0.160751671
